# Anastomotic Leakage After Colorectal Surgery: Exploratory Analysis of Perioperative Risk Factors in a 672-Patient Cohort

**DOI:** 10.7759/cureus.103531

**Published:** 2026-02-13

**Authors:** Timur Buniatov, Cornelia Weidinger, Christian Krautz, Maximilian Brunner, Roland C E Francis, Robert Gruetzmann, Matthias Maak

**Affiliations:** 1 Department of General and Visceral Surgery, University Hospital Erlangen, Friedrich-Alexander-Universität Erlangen-Nürnberg, Erlangen, DEU; 2 Department of Anesthesiology, University Hospital Erlangen, Friedrich-Alexander-Universität Erlangen-Nürnberg, Erlangen, DEU; 3 Department of Anesthesiology, Dubai Hospital, Dubai Academic Health Corporation, Dubai, ARE; 4 Department of Surgery, University Hospital Erlangen, Friedrich-Alexander-Universität Erlangen-Nürnberg, Erlangen, DEU; 5 Department of General and Visceral Surgery, Kreiskrankenhaus St. Anna Höchstadt an der Aisch, Höchstadt a. d. Aisch, DEU

**Keywords:** anastomotic leakage, colorectal surgery, hypothermia, intraoperative blood loss, intraoperative management, intravenous fluids, norepinephrine, operative time, perioperative care

## Abstract

Background

Anastomotic leakage (AL) is a serious complication of colorectal surgery, associated with re-intervention, prolonged hospitalization, and impaired long-term outcomes. Although patient- and procedure-related risk factors are well established, the impact of perioperative conditions, thermal management, fluid balance, intraoperative blood loss, and vasopressor-supported hemodynamics remains incompletely defined, with heterogeneous and confounded evidence.

Methods

In this retrospective single-center cohort study, 672 colorectal procedures with primary anastomosis at the University Hospital Erlangen (2013-2018) were analyzed. The primary endpoint was anastomotic leakage within 30 days, defined by clinical and/or radiologic evidence requiring intervention. Examined variables included age, sex, body mass index (BMI), ASA class, surgical urgency, operative duration, minimum intraoperative core temperature, estimated blood loss (>500 mL), total crystalloid volume (>3500 mL), and intraoperative norepinephrine administration. Associations with anastomotic leakage were evaluated using univariable and exploratory multivariable logistic regression.

Results

Anastomotic leakage (AL) occurred in 20/672 procedures (3.0%) and was associated with a markedly longer hospital stay (41.5 [IQR 31.3-60.3] vs. 11 [IQR 9.0-17.0] days; p < 0.001). In an exploratory multivariable logistic regression, ASA class III/IV (OR 3.8, 95% CI 1.3-11.3; p = 0.016) and operative duration ≥300 min (OR 3.6, 95% CI 1.2-13.6; p = 0.025) were independently associated with leakage. In contrast, intraoperative hypothermia (<35.5°C), estimated blood loss (>500 mL), and any norepinephrine use showed unadjusted associations with AL but were not independently associated after adjustment.

Conclusion

High ASA class (III/IV) and prolonged operative duration were the only independent risk factors for anastomotic leakage in this study. Intraoperative exposure variables such as hypothermia, estimated blood loss, and any norepinephrine use were not independently associated after adjustment.

## Introduction

Anastomotic leakage (AL) remains one of the most serious complications after colorectal surgery, with substantial clinical and economic consequences. Reported AL rates vary widely (approximately 2%-19%, and up to 30% in high-risk subgroups), reflecting differences in case mix, operative technique, comorbidity burden, and perioperative care, as well as ongoing heterogeneity in definitions and grading systems [1-5]. When AL occurs, it is associated with increased morbidity, re-interventions, prolonged hospitalization, and higher short-term mortality; it also impairs long-term quality of life and oncologic outcomes [6-8].

Anastomotic healing depends on adequate perfusion, minimal tension, meticulous hemostasis, and effective contamination control. Established risk factors include patient-related characteristics (e.g., comorbidities, higher ASA class, and elevated BMI), medication exposure (e.g., corticosteroids and chemotherapy), and procedure-related factors such as prolonged operative duration and emergency surgery [3,4,9-12]. In contrast, potentially modifiable intraoperative exposures, hypothermia, blood loss, vasopressor support, and fluid administration have been linked to AL less consistently, and interpretation is often limited by heterogeneity and confounding by indication [13-15]. For example, crystalloid overload has been shown to induce inflammatory changes at intestinal anastomoses in experimental work [14], whereas clinical reviews report equivocal associations for fluid and hemodynamic exposures and limited evidence for core temperature or mean arterial pressure in colorectal anastomotic leakage [15].

The primary objective of this retrospective single-center study was to explore associations between perioperative factors, particularly routinely documented intraoperative exposures, and AL in a real-world colorectal surgery cohort. A secondary objective was to provide pre-ERAS benchmark estimates of leakage incidence and practice patterns. Importantly, the pre-ERAS setting is informative beyond benchmarking because perioperative management was less protocol-driven, preserving greater real-world variation in intraoperative practices and reducing the influence of bundled pathway effects that can obscure associations with individual exposures [16].

## Materials and methods

Study design

This retrospective single-center cohort study analyzed patients who underwent colorectal surgery with creation of a primary anastomosis at the University Hospital Erlangen, Germany, between January 2013 and December 2018. Eligible participants were adults (≥18 years) undergoing elective or emergency colorectal resections for colorectal cancer or benign conditions such as diverticular disease. Patients who underwent resection without reconstruction and patients having only small bowel resection and anastomosis, as well as those with incomplete medical records, were excluded to preserve data integrity. The final analytic cohort comprised 672 procedures. Analyses were performed on a complete-case basis. Cases with missing documentation required for the primary outcome assessment or missing key covariates were not included in the respective regression analyses. Because the dataset was assembled retrospectively from routine electronic records, a reliable breakdown of excluded cases by reason could not be reconstructed from the extraction logs and is acknowledged as a limitation.

The primary outcome was anastomotic leakage (AL) within 30 days, defined as clinical and/or radiological evidence of anastomotic dehiscence or an intra-abdominal/pelvic collection communicating with the anastomosis requiring therapeutic intervention. Outcome status was abstracted from surgical notes, radiology reports, and discharge summaries.

Comprehensive patient data were extracted from electronic health records and operative/anesthesiology protocols. Baseline variables included age (years), sex, BMI (kg/m²), a dichotomized variable for obesity according to the WHO definition (BMI ≥30 kg/m²), and ASA class (I-IV).

Operative variables included the urgency of surgery (elective vs. emergency) and operative time (minutes); additionally, a dichotomized variable with a cut-off of 300 minutes for intraoperative time (≥300 vs. <300 min) was created based on previous research [4,13,15].

Intraoperative conditions recorded from anesthesia charts were minimum core temperature (°C; hypothermia predefined as <35.5°C), estimated blood loss (dichotomized at >500 mL), crystalloid load (total intraoperative crystalloids; high load predefined as >3500 mL), and norepinephrine use (any intraoperative infusion/bolus vs. none). Cut-offs were prespecified based on prior reports and pathophysiological plausibility [17-19]. Temperature was monitored per institutional routine (esophageal or bladder probes for core readings).

This retrospective study was based exclusively on anonymized routine clinical data; therefore, formal ethical approval was not required under institutional policies and local regulations. This was confirmed by the Ethics Committee of Friedrich-Alexander University (FAU) Erlangen-Nürnberg (protocol no. 26-4-ANF, 09 January 2026). The study was conducted in accordance with the Declaration of Helsinki.

Surgical technique and institutional leak management

At our institution, colorectal anastomoses were performed using standardized techniques. For right- and left-sided colectomies, an end-to-end, single-layer, interrupted handsewn anastomosis with polyglactin-910 3-0 was routinely used; polydioxanone 5-0 reinforcement sutures were added at the surgeon’s discretion. For low anterior resections, a stapled end-to-end anastomosis with 29-31 mm circular staplers was standard.

Postoperative surveillance for anastomotic leakage (AL) followed a uniform clinical pathway. In the presence of suggestive findings (fever, rising C-reactive protein, abdominal pain, or abnormal drain output), contrast-enhanced CT (with intravenous and ± rectal contrast when appropriate) was typically obtained. Flexible endoscopy was used particularly for suspected low rectal leaks.

Management strategies were aligned with international practice and tailored to the anastomotic level and patient condition. Colonic leaks generally underwent early reoperation (laparotomy or laparoscopy) with source control and diversion as indicated. For low rectal leaks, endoscopic vacuum therapy was preferred when feasible; otherwise, operative management was pursued. The final approach (including diversion) was left to the treating team based on intraoperative and patient factors.

Statistical analysis

Analyses were performed using Jamovi software (version 2.3.28; Sydney, Australia). Continuous variables are reported as median (IQR; 25th-75th percentiles), and categorical variables as n (%). Group comparisons used the Mann-Whitney U test for continuous data and the χ² test or Fisher’s exact test for categorical data, as appropriate.

Univariable logistic regression was used to assess the association of each candidate factor with anastomotic leakage (AL). Variables with p < 0.05 in univariable analysis were entered into an exploratory multivariable logistic regression to estimate independent associations. To reduce the risk of overfitting, given the low number of AL events, we applied a parsimonious model specification and report odds ratios (OR) with 95% confidence intervals (CI).

The multivariable model was specified to quantify adjusted associations rather than to develop or validate a clinical prediction tool; therefore, no internal validation procedures (e.g., bootstrapping or cross-validation) were performed. Results should be interpreted as exploratory and hypothesis-generating.

## Results

Patient characteristics and descriptive data

A total of 672 colorectal procedures with primary anastomosis were analyzed, of which 20 (3.0%) developed anastomotic leakage (AL) (Table 1). AL was associated with a markedly longer hospital stay (median 41.5 [IQR 31.3-60.3] vs. 11.0 [IQR 9.0-17.0] days; p < 0.001). Patients with AL were older than those without leakage (median 72.5 vs. 63.0 years; p = 0.004), with a higher proportion aged ≥65 years (p = 0.011) and more frequently classified as ASA III/IV (overall ASA distribution p < 0.001; ASA III/IV p < 0.001). Sex and BMI did not differ significantly between groups (female/male p = 0.865; BMI p = 0.789; obesity p = 0.133).

**Table 1:** Patient characteristics and intraoperative factors according to anastomotic leakage. ASA = American Society of Anesthesiologists; BMI = body mass index; IQR = interquartile range. Percentages are shown per column (within-group). Values are presented as median (IQR; 25th–75th percentiles) or n (%), as appropriate. P-values were calculated using the Mann–Whitney U test for continuous variables and the chi-square test for categorical variables, as appropriate. The test statistic column reports U for Mann–Whitney and χ² for chi-square tests. For multi-category variables (gender, ASA class), the test statistic and p-value refer to the overall comparison between groups; category counts are shown in the rows below.

	All patients (n=672)	No Leakage (n=652)	Leakage (n=20)	Test statistic (U or χ²)	p-value
Baseline characteristics					
Age (years), median (IQR)	64.0 (52.0–73.0)	63.0 (52.0–73.0)	72.5 (66.3–82.0)	U = 4027	0.004
Age ≥65 years, n (%)	316 (47.0)	301 (46.2)	15 (75.0)	χ² = 6.48	0.011
Gender, n (%)	—	—	—	χ² = 0.0291	0.865
Female, n (%)	315 (46.9)	306 (46.9)	9 (45.0)	—	—
Male, n (%)	357 (53.1)	346 (53.1)	11 (55.0)	—	—
ASA class (I–IV), n (%)	—	—	—	χ² = 31.6	<0.001
ASA I, n (%)	66 (9.8)	66 (10.1)	0 (0.0)	—	—
ASA II, n (%)	409 (60.9)	403 (61.8)	6 (30.0)	—	—
ASA III, n (%)	191 (28.4)	179 (27.5)	12 (60.0)	—	—
ASA IV, n (%)	6 (0.9)	4 (0.6)	2 (10.0)	—	—
ASA III/IV, n (%)	197 (29.3)	183 (28.1)	14 (70.0)	χ² = 16.5	<0.001
Obesity (BMI ≥ 30 kg/m²), n (%)	144 (21.4)	137 (21.0)	7 (35.0)	χ² = 2.25	0.133
Hospital stay (days), median (IQR)	11 (9.0–17.0)	11 (9.0–17.0)	41.5 (31.3–60.3)	U = 2207	<0.001
Risk factors					
Emergency surgery, n (%)	61 (9.1)	58 (8.9)	3 (15.0)	χ² = 0.876	0.349
Operative duration ≥ 300 min, n (%)	63 (9.4)	57 (8.7)	6 (30.0)	χ² = 10.3	0.001
Intraoperative hypothermia <35.5 °C, n (%)	83 (12.4)	77 (11.8)	6 (30.0)	χ² = 5.93	0.015
Intraoperative blood loss >500 mL, n (%)	82 (12.2)	76 (11.7)	6 (30.0)	χ² = 6.09	0.014
High intraoperative crystalloid load (>3500 mL), n (%)	297 (44.2)	284 (43.6)	13 (65.0)	χ² = 3.62	0.057
Norepinephrine use, n (%)	172 (25.6)	162 (24.8)	10 (50.0)	χ² = 6.45	0.011

Univariable analysis

Emergency surgery was performed on 61 patients (9.1%) and was not associated with AL (p = 0.349). On univariable analysis, operative duration ≥ 300 minutes (p = 0.001), intraoperative hypothermia < 35.5°C (p = 0.015), blood loss > 500 mL (p = 0.014), and norepinephrine use (p = 0.011) were associated with AL, while a high crystalloid load (> 3500 mL) showed a non-significant trend (p = 0.057) (Tables 1, 2).

**Table 2:** Multivariable logistic regression analysis of risk factors for anastomotic leakage. Values are presented as odds ratios (OR) with 95% confidence intervals (CI) from a multivariable logistic regression model. Norepinephrine use indicates any intraoperative administration.

Risk Factors	OR	95% CI	p-value
Age ≥ 65 years	2.1	0.7 – 6.4	0.181
ASA III/IV	3.8	1.3 – 11.3	0.016
Intraoperative blood loss >500 mL	1.4	0.5 – 4.5	0.526
Operative duration ≥ 300 min	3.6	1.2 – 13.6	0.025
Intraoperative hypothermia <35.5 °C	2.0	0.7 – 5.7	0.193
Norepinephrine use	1.3	0.5 – 3.4	0.636

Multivariable analysis

In an exploratory multivariable logistic regression (Table 2), ASA class III/IV (OR 3.8, 95% CI 1.3-11.3; p = 0.016) and operative duration ≥300 min (OR 3.6, 95% CI 1.2-13.6; p = 0.025) remained independently associated with AL. In contrast, hypothermia <35.5°C (OR 2.0, 95% CI 0.7-5.7; p = 0.193), blood loss >500 mL (OR 1.4, 95% CI 0.5-4.5; p = 0.526), and any norepinephrine use (OR 1.3, 95% CI 0.5-3.4; p = 0.636) were not independently associated with AL. Age ≥65 years likewise did not reach statistical significance in this model (OR 2.1, 95% CI 0.7-6.4; p = 0.181).

## Discussion

In this retrospective single-center cohort of 672 colorectal procedures with primary anastomosis, anastomotic leakage (AL) occurred in 3.0% of cases and was associated with a fourfold increase in length of stay (41.5 vs. 11 days), underscoring its disproportionate clinical burden even at low incidence.

Following multivariable adjustment, only two factors remained independently associated with AL: higher ASA classification (III/IV) and operative duration ≥300 min.

Several intraoperative exposures showed unadjusted associations with AL but did not reach statistical significance after adjustment. Given the low number of events (n = 20), correlations among intraoperative variables, and residual confounding, these findings should be interpreted cautiously and not as evidence that clinically relevant effects are absent. In this setting, confounding by indication may lead intraoperative exposures to reflect underlying illness severity and case complexity rather than causal effects, while the wide confidence intervals primarily indicate limited statistical power and imprecision.

The observed AL rate lies at the lower end of previously reported ranges [1,6]. This may reflect institutional practice patterns in a high-volume university center with a colorectal surgery focus.

This analysis was conducted as a real-world benchmark in a pre-ERAS cohort to assess whether previously reported intraoperative associations are detectable under routine standard care prior to widespread implementation of enhanced recovery pathways.

ASA and operative time: independent predictors

In our cohort, ASA class III/IV (OR 3.8, p = 0.016) was one of the two independent predictors, underscoring the importance of baseline physiological reserve. A high ASA score reflects the cumulative burden of cardiopulmonary disease, diabetes, vascular pathology, and frailty, all of which may compromise tissue perfusion and immune response. Multiple systematic reviews and registry studies have reported higher AL rates in patients with ASA ≥3, corroborating our finding and highlighting the need for preoperative risk stratification and, in selected cases, a lower threshold for a protective stoma formation [3,12]. Older age was associated with AL in univariable analysis (≥65 years, p = 0.011) but did not reach statistical significance in the multivariable model, consistent with age serving as a proxy for reduced physiological reserve and comorbidity burden captured by ASA class and correlated perioperative factors. Older age has likewise been reported in prior work [20].

Prolonged operative duration (surgery duration ³ 300 min) emerged as the second independent risk factor of AL (OR 3.6, p=0.025), supporting the concept that time functions as a surrogate marker for procedural complexity. Longer operations typically reflect one or more unfavorable intraoperative conditions: difficult exposure in obese or previously operated patients, dense adhesions, unexpected findings, intraoperative contamination, episodes of hypotension or bleeding, and technically demanding pelvic dissection. Each of these factors may increase tissue trauma and edema, obscure anatomical planes, and hinder precise, tension-free, layer-to-layer anastomosis construction. Several large series have shown a stepwise increase in AL once operative time exceeds approximately 180-200 minutes, with substantially higher odds beyond 240-300 minutes, which is in line with our findings [4,13,15].

However, operative time is an aggregate metric that condenses diverse mechanisms into a single value. It cannot differentiate between additional minutes spent on meticulous hemostasis and quality control versus time lost to repeated dissection, uncontrolled bleeding, or prolonged hypotension. Future work should therefore move beyond duration alone and incorporate mechanistic intraoperative variables that may mediate the link between time and AL, including perfusion-related measures such as fluorescence angiography [21]; markers of inflammatory and tissue-trauma load such as contamination, cumulative blood loss, and transfusions [22]; and structured indicators of technical complexity such as low rectal anastomosis, multiple stapler firings, multivisceral resection, or conversion to open surgery [23].

Our data support using operative duration as a pragmatic screening surrogate for high-risk cases, but also underlines its limitations. Given the current paucity of detailed physiological and technical intraoperative data in routine practice, operative time should be interpreted as a useful “alarm bell” rather than a causal factor. When surgery substantially exceeds the expected duration, the team should assume an increased AL risk and respond with heightened vigilance, for example, by reassessing anastomotic perfusion, critically reviewing the anastomosis, lowering the threshold for a protective stoma, and ensuring intensified postoperative monitoring and early leak detection.

Intraoperative exposures: unadjusted signals not confirmed after adjustment

Intraoperative hypothermia (<35.5°C), estimated blood loss (>500 mL), any norepinephrine administration, and higher crystalloid volumes showed unadjusted associations with AL; however, none remained independently associated after multivariable adjustment. Bleeding may impair anastomotic construction by reducing visibility and increasing tissue manipulation and may also compromise healing through hypovolemia and reduced oxygen delivery, mechanisms that are biologically plausible in the context of anastomotic integrity. Several studies have reported an association between increased intraoperative blood loss and AL risk [24,25].

Intraoperative hypothermia can adversely affect coagulation and peripheral perfusion and has been linked to broader postoperative morbidity [19,26,27]. However, evidence specifically addressing hypothermia as a risk factor for colorectal AL remains limited and heterogeneous [28].

Similarly, norepinephrine use is challenging to interpret causally because it often reflects underlying hemodynamic instability (e.g., hypovolemia, bleeding, or cardiac dysfunction). Nevertheless, some studies have suggested a potential association between vasopressor use and AL [13].

From a clinical perspective, our findings reinforce that global patient condition (ASA class) and procedural complexity (operative duration) are robust signals in routinely available data and may serve as pragmatic triggers for heightened intraoperative and postoperative vigilance. From a research perspective, this cohort provides a pre-ERAS benchmark of leakage incidence and perioperative practice patterns, allowing contemporary outcomes to be interpreted within an institutional and temporal context.

Strengths and limitations

This study has several strengths, including a comparatively large single-center cohort (n = 672) with comprehensive anesthesia records, enabling the joint assessment of routinely recorded intraoperative exposures alongside patient- and procedure-related factors within a consistent institutional surgical and anesthetic framework. Anastomotic leakage was uniformly defined within 30 days using clinical and radiologic criteria. Analyses were pre-specified and physiologically grounded, and both univariable and multivariable logistic regression results are reported with effect sizes and 95% confidence intervals. Several limitations should also be acknowledged.

First, the number of AL events was low (n = 20). Together with correlations among intraoperative exposures and the likelihood of residual confounding, this limits statistical power and increases the risk of model instability; therefore, the multivariable analysis should be interpreted as exploratory and hypothesis-generating rather than conclusive. No internal validation or calibration was performed. Outcome severity was not stratified, and management pathways such as reoperation versus endoscopic or interventional treatment could not be reliably captured from routine documentation, which limits clinical granularity.

Second, residual confounding is likely because key anatomical and technical variables, such as anastomotic height, diverting stoma, surgeon experience, neoadjuvant therapy, transfusion exposure, and stapling technique, were not available and may have influenced both exposure patterns and leakage risk. Surgeon- or team-level adjustment was not possible, and clustering of higher-risk cases within specific operators or subspecialist teams cannot be excluded. Because this was a retrospective, complete-case analysis, the exact number and reasons for excluded cases could not be reliably reconstructed.

Third, measurement limitations apply to several variables. Estimated blood loss derived from anesthesia records may be imprecise (e.g., due to irrigation), crystalloid totals did not capture colloids or blood products, and vasopressor use was documented without dose-time exposure or cumulative metrics. Postoperative weight change, a clinically relevant proxy for fluid overload, was also not available. Taken together, these limitations underscore the exploratory nature of this work. While the observed adjusted associations are internally plausible, generalizability is limited, and the findings should primarily be interpreted as benchmark evidence to inform future adequately powered prospective investigations.

Future research

The present findings highlight the importance of studying potentially modifiable intraoperative exposures with greater methodological precision. Intraoperative variables such as blood pressure, temperature, vasopressor support, and fluid administration are dynamic over time, frequently co-occur, and are strongly influenced by patient condition and procedural complexity. Future research should therefore prioritize prospective, multicenter studies with standardized definitions of anastomotic leakage and sufficient granular perioperative data to clarify whether intraoperative management contributes independently to leakage risk.

To improve interpretability, future datasets should move beyond single thresholds or isolated time-point values and incorporate time-resolved exposure metrics, including time-weighted mean arterial pressure (“hypotension dose”), depth and duration of hypothermia, vasopressor dose-time exposure (e.g., norepinephrine area-under-the-curve), transfusion exposure and net fluid balance (including blood products), as well as postoperative weight change as a pragmatic marker of fluid overload. These physiological measures should be complemented by key anatomical and technical variables (e.g., anastomotic height, diverting stoma, stapler firings, conversion, multivisceral resection) and, where available, objective perfusion assessment such as ICG fluorescence angiography [29]. Advanced analytical approaches, including artificial intelligence and machine learning, may further support the integration of high-dimensional, time-resolved information from anesthesia records, hemodynamic waveforms, perfusion imaging, and operative documentation, potentially enabling dynamic and individualized risk assessment [30]. However, such tools should be viewed as adjunctive to surgical and anesthetic judgment and must be externally validated and prospectively evaluated for real-world usability, interpretability, fairness, and clinical impact before broader implementation.

## Conclusions

ASA class III/IV and operative duration ≥300 minutes were independently associated with an increased risk of anastomotic leakage in this retrospective cohort. Hypothermia, higher estimated blood loss, and norepinephrine use were associated with leakage in unadjusted analyses but did not remain statistically significant after adjustment. Within the constraints of this exploratory retrospective model, these findings should inform risk awareness and benchmarking rather than prompt protocol changes. Larger multicenter prospective studies are required to determine whether these potentially modifiable factors have independent effects on leakage risk and to guide prevention efforts.
